# Supplementary material for: Pain Control Affects the Radiographic Diagnosis of Segmental Instability in Patients with Degenerative Lumbar Spondylolisthesis
Source: J Clin Med. 2021 Sep 2;10(17):3984. doi: 10.3390/jcm10173984 (PMC8432229; doi:10.3390/jcm10173984)
Supplement: Supplementary file 1 [file jcm-10-03984-s001.zip › jcm-1343748-supplementary.pdf]

Table S1. Investigation of segmental instability by study

	This study	Jarzem et al. [16]	Lilius et al.[17]	Williams et al.[18]
**Number of patients**	100	50	67 (109)[&]	40[ψ]
**Age (range)**	53.9 (28-69)	U (18-70)	44 (19-64)	40 (18-55)
**Methods of intervention**	IM analgesia (ketolorac 30mg)	Transcutaneous electrical nerve stimulation	Facet joint injection	Self-administer oral analgesia
**Duration demand**	30 minutes	Within one hour	Within one hour	45-60 minutes
**Assessment**				
**Pain score**	Average 34.7 mm reduction	U	Average 18.3 mm reduction	Around 23-30 mm reduction
**Spinal motion**	Radiographic Flexion/extension	Gravity goniometer	Spine curvature measurement[◎]	Spine curvature measurement[Φ]
**Effect of intervention**	Increased dynamic lumbar lordosis by 9.17% and slip percentage in motion segments by 6.65%.	Increase in ROM during flexion and extension as measured by an inclinometer.	Pain relief did not change flexion extension ROM	Pain reduction did not result in an gain in lumbar curvature

IM = intramuscular injection; U = un-mentioned; & = 42 patients received placebo injection (normal saline);
ψ = including 20 acute and 20 chronic low back pain participants; ◎ = skin marker method; Φ= Fiber-optic base reference.
